# Exploiting PLGA-Based Biocompatible Nanoparticles for Next-Generation Tolerogenic Vaccines against Autoimmune Disease

**DOI:** 10.3390/ijms20010204

**Published:** 2019-01-08

**Authors:** Giuseppe Cappellano, Cristoforo Comi, Annalisa Chiocchetti, Umberto Dianzani

**Affiliations:** 1 Department of Health Sciences, Università del Piemonte Orientale, 28100 Novara, Italy; annalisa.chiocchetti@med.uniupo.it (A.C.); umberto.dianzani@med.uniupo.it (U.D.); 2 Interdisciplinary Research Center of Autoimmune Diseases, Università del Piemonte Orientale, 28100 Novara, Italy; cristoforo.comi@med.uniupo.it; 3 Center for Translational Research on Autoimmune and Allergic Disease—CAAD, Università del Piemonte Orientale, 28100 Novara, Italy; 4 Department of Translational Medicine, Università del Piemonte Orientale, 28100 Novara, Italy

**Keywords:** nanoparticle, PLGA, tolerogenic vaccination, inverse adjuvant

## Abstract

Tolerogenic vaccines are aimed at inhibiting antigen-specific immune responses. Antigen-loaded nanoparticles (NPs) have been recently emerged as ideal tools for tolerogenic vaccination because their composition, size, and capability of loading immunomodulatory molecules can be readily exploited to induce peripheral tolerance. Among polymeric NPs, poly(lactic-co-glycolic acid) (PLGA) NPs have the advantage of currently holding approval for several applications in drug delivery, diagnostics, and other clinical uses by the Food and Drug Administration (FDA). PLGA-NPs are non-toxic and display excellent biocompatibility and biodegradability properties. Moreover, surface functionalization may improve their interaction with biological materials, thereby optimizing targeting and performance. PLGA-NPs are the most extensively studied in pre-clinical model in the field of tolerogenic vaccination. Thus, this review describes their potential applications in the treatment of autoimmune diseases.

## 1. Immunogenic and Tolerogenic Vaccinations

The immune response is normally triggered only by dangerous antigens, where the danger (i.e., pathogen) is recognized because of its ability to induce inflammation either directly or indirectly by inducing tissue necrosis. Thus, pathogens activate effector lymphocytes, which in turn support inflammation to eliminate these invaders. After this initial phase, effector lymphocytes are for the most part eliminated with the exception of an expanded set of long-lived memory lymphocytes that confers immunological memory ensuring a rapid immune response following re-exposure to the same pathogen. 

**Figure 1:**
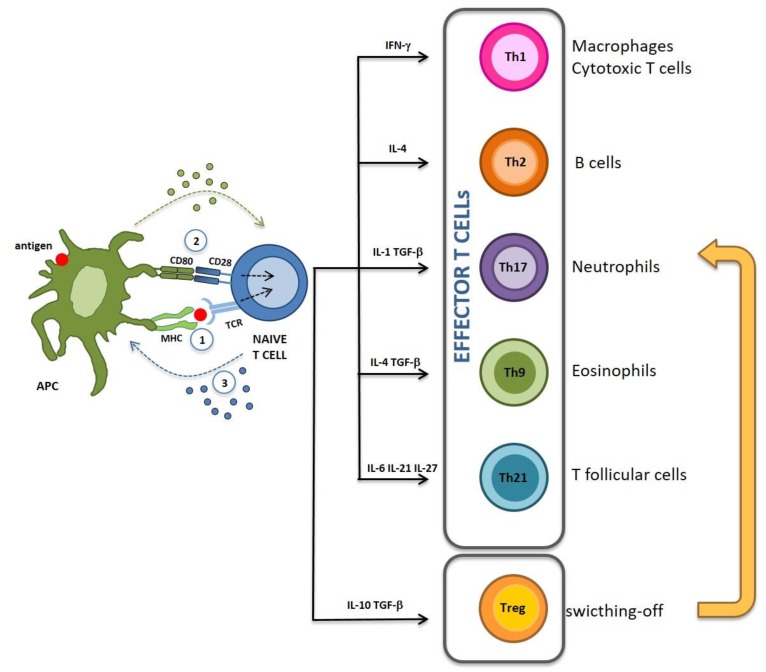
Activation of immune response. Upon activation by APC, naïve T cell depending on the cytokine milieu. are polarized into different T effector cell subsets (Th1, Th2, Th17, Th9, Th21) and into Treg cells, that will switch-off the antigen-specific effector response. Green and blue dotted arrows indicate signal 2 and signal 3.

T-cell activation is critical for the initiation and regulation of the immune response, it involves interaction with an antigen presenting cell (APC) and needs three signals (Figure 1). Signal 1 is delivered by the interaction of the T-cell receptor (TCR) with the antigen presented on major histocompatibility complex (MHC) molecules expressed by APC. Signal 2 is mediated by the engagement of co-stimulatory molecules such as B7.1 (CD80) and B7.2 (CD86) on APC and CD28 on the T cell. These two signals start a cross-talk between T cells and APCs which both release cytokines that collectively will define the inflammatory milieu (signal 3). According to it, T cells can differentiate into different types of cytokine-secreting cells [1,2]. The presence of interferon (IFN)-γ drives differentiation of T helper (Th) type 1 (Th1) cells, which mainly secrete IFN-γ and interleukin (IL)-2, and support activation of macrophages, natural killer (NK), and cytotoxic T lymphocytes [3]; IL-4 drives differentiation of Th2 cells which mainly secrete IL-4 and support B cell activation and antibody production [4]. In the absence of IFN-γ and IL-4, transforming growth factor (TGF)-β and IL-1 drive differentiation of Th17 cells, which mainly secrete IL-17 and support the neutrophil response [5]. When both TGF-β and IL-4 are present, CD4+T helper cells tend to differentiate into Th9 cells which are potent secretors of IL-9, thereby supporting the eosinophil response [6,7]. IL-6, IL-21 and IL-27 drives differentiation of T follicular helper (TFH) cells, which secrete IL-21, thus supporting B cell activation and germinal center formation [1]. Lastly, the presence of IL-10 drives differentiation of regulatory T (Treg) cells, which produce IL-10 and TGF-β, two potent inhibitors of the inflammatory and immune response [8]. Effector T cells elicit an aggressive response toward pathogens and cancer cells while Treg cells switch this response off.

Classic immunogenic vaccines induce an effector immune response and elicit immunological memory without the need for experiencing prior infection. They are composed of two components: (1) the antigen, conferring immune response specificity; and (2) the adjuvant, which has the role of keeping the vaccine into the injection site, known as the “depot effect”, while triggering inflammation, necessary to promote the adaptive immune response. Specifically, adjuvants are known to activate T cells by: (1) increasing MHC-II expression thus enhancing antigen presentation APCs, which then trigger *signal 1*; (2) inducing APCs to express costimulatory ligands able to trigger *signal 2*; (3) inducing secretion of cytokines capable of directing the immune response toward appropriate effector functions (*signal 3*) (Figure 1).

In contrast to immunogenic vaccines eliciting immune responses against a pathogen, tolerogenic vaccines, also known as inverse vaccines, are aimed to suppress the pathological immune response to both allergens and autoantigens. A key role in suppression of the immune response is played by Treg cells that include natural Treg (nTreg) cells, which differentiate in the thymus and mainly recognize autoantigens, and induced Treg (iTreg) cells, which differentiate in the periphery from different types of effector Th cells. Tregs exert their activity through several mechanisms, including secretion of anti-inflammatory cytokines, such as IL-10 and TGF-β [9,10], and metabolic disruption and modulation of dendritic cell (DC) maturation and function [11]. Decreased numbers and defective function of Tregs have been detected in several inflammatory and autoimmune diseases [12]. Thus, the efficacy of several tolerogenic vaccines depends on the activation, expansion, and differentiation of antigen-specific Tregs generating a long-lasting antigen memory (dominant tolerance). Notably, Tregs can directly suppress several types of effector T cells [11] and can also inhibit APC function as attested by intravital microscopy experiments showing Tregs interaction with DC-bearing antigen prior to effector T cell function inhibition [13]. In addition to dominant tolerance, tolerogenic vaccines can also induce a passive or deletional tolerance leading to apoptosis or anergy of autoreactive T cells [14].

Given that high dose vaccines, intravenous and mucosal routes of administration, prolonged vaccine exposure, and the presence of IL-10 and TGF-β are well established tolerogenic determinants, tolerogenic vaccine-induced peripheral tolerance strictly depends on antigen dose [15,16], delivery route [17], exposure time to the antigen [18] and cytokine milieu [19].

**Figure 2:**
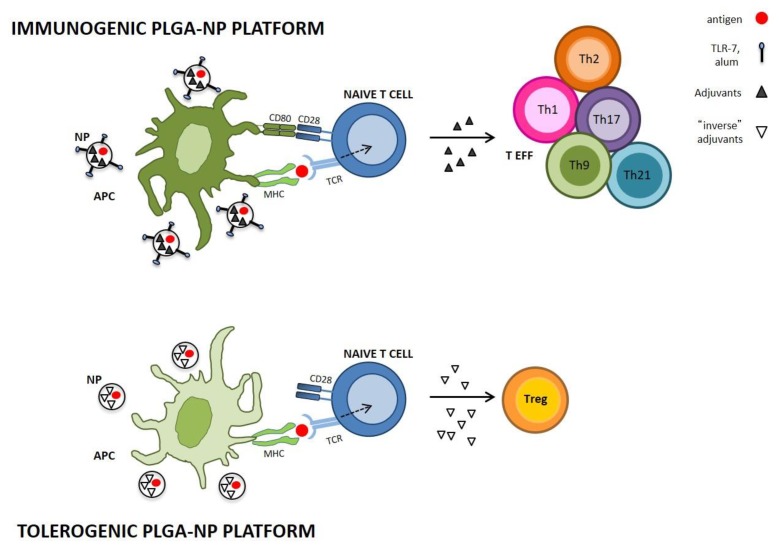
Immunogenic and tolerogenic PLGA-NP platforms. Immunogenic PLGA-NPs may include standard adjuvants such as alum or Toll-like receptor (TLR-7) agonists, capable to activate APC and promote naïve T cell differentiation into effector cell (TEFF); on the contrary, in the absence of costimulatory signals, tolerogenic PLGA-NPs by employing “inverse adjuvants” induce tolerogenic APC that leads to the expansion of antigen-specific Treg. Dotted arrow indicates activation of naïve T cells and solid arrow indicates its differentiation into TEFF or Treg.

Based on this paradigm, one of the most promising approaches is represented by nanoparticles (NPs)-based vaccination. In particular, the use of NPs has greatly helped to finely tune the immunogenicity of vaccines. On the one hand, when used for immunogenic vaccination, NPs can be designed to improve antigen stability and modulate activation of innate immunity in order to elicit optimal immune responses with minimal toxicity. On the other hand, in the case of tolerogenic vaccination, NPs can be used as platforms to modulate the doses and times of delivery not only of the antigens but also of those substances needed to promote tolerance (tolerogenic adjuvants) [20] (Figure 2).

## 2. Nanoparticles: An Overview

Nanomedicine holds great promise for developing new and more effective medical treatments [21,22]. In the last decade, research interest in micro/nanoparticles has rapidly increased, especially in the area of drug delivery, due to the availability of several innovative tools such as micelles, liposomes, silver and gold particles, nanocrystals, dendrimers, nanotubes, lipid NPs, and polymeric NPs, all employed as carriers for various bioactive molecules [23,24,25,26,27,28]. 

NPs stabilize and protect the drug from degradation and improve drug targeting to specific tissues, thereby increasing drug efficacy and reducing side effects [29]. A large number of studies have focused on anti-neoplastic drugs as many types of NPs passively increase cancer targeting due the “enhanced permeability and retention” (EPR) effect, which exploits the wide fenestrations of the angiogenic vasculature [23,30]. Moreover, NPs have been used to deliver DNA vectors, clinical imaging probes, and vaccine components [31,32,33,34].

Key points for their use in vaccination are their capacity to interact with several types of immune cells and encapsulate/release antigens and other immunomodulatory molecules to influence the host immune response [35]. The extent of the interaction between NPs and immune cells is determined by several intrinsic NP parameters such as size, shape, chemical composition, surface properties, charge density (i.e., zeta potential), and oxidative potential [35]. The influence of antigen-loaded NPs on the immune response can be finely modulated by changing their composition, size, and loading capacity of immunomodulatory molecules [36]. Importantly, all these features have been shown to play a role in determining either activation or shutdown of the immune response while performing immunogenic or tolerogenic vaccination, respectively.

A key characteristic of NPs that makes them particularly suited for immunogenic vaccination is the type of nanomaterial that has been used for their production. Indeed, the properties of the various nanomaterials available can significantly enhance the immune response through direct and/or indirect (i.e., cell toxicity) activation of inflammatory cells and APCs [37]. For instance, cationic (positively charged) NPs tend to induce higher inflammatory reactions than anionic (negatively charged) or neutral NPs [37,38,39,40]. Another important feature of NP-based immunogenic vaccines is represented by the size of the NP being used, which can influence biodistribution, APC uptake, and the type of Th cell responses. For example, small particles (<50 nm), which rapidly move to the lymph nodes and are therefore more efficiently captured by APCs, are deemed the best choice for optimal antigen presentation [41]. By contrast, large particles (>0.5–1 µm), which tend to remain in the proximity of the injection site and are therefore poorly captured by APCs, are better carriers for compounds that can either support and modulate the immune response to immunogenic vaccines, as in the case of adjuvants, or trigger tolerance to tolerogenic vaccines, as in the case of inverse adjuvants [39,42]. Moreover, large particles tend to induce Th1, whereas small particles tend to induce Th2 responses [43].

Particular interest has been recently focused on polymeric NPs made of several biocompatible and biodegradable materials such as poly (lactic-co-glycolic acid) (PLGA)-NPs, which have been widely used to increase potency and bioavailability of several drugs and currently hold FDA approval for several therapeutic applications [44]. PLGA is a biodegradable polymer widely used in surgical sutures, bone plates, and orthopedic implants. In particular, PLGA microspheres have been used as long-acting drug delivery system since 1984. One of the key advantages of using PGLA-NPs is that they can be easily loaded with a wide variety of molecules. The degradation kinetics of NPs and the release rate of the encapsulated molecules vary depending on PGLA physicochemical properties such as lactide to glycolide ratio, molecular weight, crystal profile, storage temperature and surface coating materials [44,45,46,47]. PLGA-NPs composed of high amounts of lactic acid are soluble in dichloromethane, chloroform or acetone, while those composed of high amounts of glycolic acid are soluble in fluorinated solvents, such as hexafluoroisopropanol [44,46]. Low amounts of lactic acid and low molecular weight of PLGA decrease the glass transition temperature, which is usually above the physiological temperature for PLGA [48,49]. PLGA-NPs size (evaluated by dynamic laser scattering-DLS [50]) and morphology (evaluated by scanning electron microscopy-SEM, transmission electronic microscopy-TEM or atomic force microscopy-AFM [51]) are influenced by several synthesis parameters. For instance, the single solvent evaporation method leads to PLGA-NPs in the 1–10 μm range, whereas the double solvent method leads to PLG-NPs in nanometer scale [52]. Moreover, surface coating with poly(ethylene glycol) (PEG) makes PLGA-NPs suitable for photodynamic applications [53]. In addition, because of its excellent photostability, PLGA-NPs may be a biodegradable substitute of quantum dots in bioimaging [54]. Because of its nontoxicity and excellent biocompatibility and biodegradability, PLGA-NPs have been the most extensively studied among polymeric NPs. 

This review will describe the use of PLGA-NPs for innovative generation of tolerogenic vaccines for treatment of autoimmune disease.

## 3. PLGA-NPs: In Vitro and In Vivo Antigen Release

As previously mentioned, the release of antigens from PLGA-NPs can be modulated by varying the ratio of lactide to glycolide used for the polymerization and/or the molecular weight (MW) of the polymers. For example, 85:25 PLGA copolymers (lactate:glycolate) show a slower release rate of the entrapped molecules than 50:50 PLGA copolymers [44,55,56]. PLGA-NPs are usually manufactured using a single or double solvent evaporation method, where poly/vinyl alcohol (PVA) is the commonly used surfactant [57,58]. 

In vitro, PLGA-NPs loaded with an antigen show a bi-phasic release kinetics characterized by an initial burst release followed by a slower and more persistent release [59]. The initial burst release depends on the molecular features and concentration of the encapsulated antigen as well as on the polymer hydrophobicity [60]. It corresponds to the rapid release of the amount of antigen bound or close to the NP surface, which dissolves in the initial water entering the polymer matrix [61]. The second phase parallels a progressive slow release of soluble PLGA oligomers and monomers from the degrading polymer [62]. Antigen release slows down with the increase in NP size because of the increased diffusion lengths in larger nanoparticles [63]. Degradation of PLGA-NPs in vitro occurs through a bulk erosion mechanism [64] consisting of three phases. In the first phase, there is a decrease of molecular weight of the polymer due to the cleavage of the ester bonds, without loss of polymer mass. In the second phase, the microenvironment acidification causes polymer mass loss due to formation of oligomers. In the third phase, the oligomers are fragmented in monomers, leading to complete solubilization of the polymer [65].

Similar to the in vitro situation, PLGA-NPs are degraded in vivo through hydrolysis of the ester bonds, but this process is accelerated in this setting because tissue cells recognize the PLGA-NPs as foreign particles and react against them by releasing enzymes and free radicals promoting PLGA degradation [66]. Moreover, PLGA oligomers display increased solubility in the blood compared to the in vitro medium [67,68]. Lastly, cells can also directly contribute to PLGA-NP degradation by phagocytizing small PLGA-NPs and hydrolyzing them to produce lactic acid and glycolic acid, then eliminated through the Krebs cycle [69]. 

PLGA-NPs can be loaded with a fluorescent dye in order to monitor the release and biodistribution of the encapsulated molecules into target tissues and organs [33,70] as well as the degradation occurring in vivo. In mice, intravenous (i.v.) injection of fluorescent PLGA-NPs of two different sizes (200 nm and 500 nm) showed that both types of NPs display the highest deposition levels in the liver followed by the spleen and the lungs. Notably, small NPs are degraded faster than the large ones in the spleen and the liver [71,72]. A similar approach showed that small PLGA-NPs can also reach the brain [58].

### 3.1. PLGA-NPs in Immunogenic Vaccination

PLGA has an intrinsic adjuvant activity, which can increase the potency of immunogenic vaccines [73]. A pioneer study in mice showed that a single subcutaneous (s.c.) injection of ovalbumin (OVA) encapsulated into PLGA-NPs is more immunogenic than soluble OVA, as shown by the production of higher serum levels of anti-OVA immunoglobulins (IgG) [74]. This finding was confirmed by other studies where encapsulation of bovine serum albumin (BSA) into PLGA-NPs increased the strength of NP-mediated vaccination as judged by enhanced anti-BSA IgG levels. This vaccination was also found to be less cytotoxic than vaccination with BSA using complete Freund’s adjuvant (CFA) [75,76,77]. Thus, it is evident that PLGA-NPs possess an intrinsic adjuvant activity due to sustained antigen release and enhanced uptake by DCs, involved in T cell activation. Moreover, PLGA-NPs are able to increase the expression of MHC class II molecules and co-stimulatory receptors (e.g., CD80 and CD86) in DCs as well as to enhance DC maturation and secretion of proinflammatory cytokines such as TNF-α and IL-1β both in vitro and in vivo [78,79,80,81,82,83,84,85], which are key factors for T cell activation. To enhance this activity, standard adjuvants, such as alum or Toll-like receptor (TLR) agonists, capable to activate DCs, have been included in the PLGA-NPs formulation [86,87,88].

### 3.2. PLGA-NPs in Tolerogenic Vaccination

The sustained antigen and immunomodulatory molecule release by PLGA-NPs makes these carriers a powerful tool for inverse vaccination despite their intrinsic adjuvant activity. Indeed, this intrinsic adjuvant activity can be counteracted by loading PLGA-NPs with anti-inflammatory molecules (either cytokines or drugs), which consequently act as *inverse adjuvants* capable of inducing tolerance. Moreover, modulating the kinetics of persistent release of the antigen makes it possible to finely tune the balance between effector and regulatory T cells. Indeed, transient exposure to antigens mimics microbial infections (non-self), thus inducing an effector T cell response, whereas persistent exposure to antigens mimics self-antigen exposure, thereby inducing a Treg-response [89].

To date, the efficacy of PLGA-NP-based tolerogenic vaccination has been demonstrated by our group [58], and others in experimental models of the following autoimmune diseases: multiple sclerosis (MS) [58,90,91,92,93,94,95,96], rheumatoid arthritis (RA) [97,98], and type 1 diabetes (T1D) [99,100,101].

#### 3.2.1. Experimental Autoimmune Encephalomyelitis

MS is an autoimmune disease targeting the myelin sheaths of the central nervous system (CNS), and it is mainly ascribed to autoreactive T cells. The most common form of the disease is characterized by a relapsing/remitting (RR) course, which generally switches to a chronic progressive course several years after the onset; the minority of patients display primary chronic progressive course without the RR phase [102]. 

Experimental autoimmune encephalomyelitis (EAE) is a widely used animal model of MS because it shares several features with the human disease, including neurological dysfunction and perivascular inflammation in the CNS [103]. Several aspects of the role of the immune response in human MS have been ascertained thanks to the results obtained in this model. EAE can be induced in several mammalian species by immunizing animals with CNS homogenate or myelin proteins, such as myelin-oligodendrocyte glycoprotein (MOG), myelin basic protein (MBP), and proteolipid protein (PLP), or using small peptides derived from these proteins [104]. The use of different immunization protocols and genetic backgrounds allows to mimic either the RR or the progressive course.

Our group has developed PLGA-NPs loaded with either the immunodominant 35–55 epitope of MOG (MOG_35–55_) in C57BL/6 mice or IL-10, used as inverse adjuvant, for prophylactic and therapeutic treatment of a chronic progressive model of EAE [58]. We selected 65:35 PLGA-NPs because they slowly release the loaded molecule for several weeks and display minimal cell toxicity along with low intrinsic adjuvant activity. Moreover, we have shown that these PLGA-NPs loaded with IL-10 completely lose their ability to induce secretion of TNF-α in vitro in peripheral blood mononuclear cells. Upon s.c. injection of these PLGA-NPs loaded with either MOG_35–55_ (PLGA-MOG) or IL-10 (PLGA-IL-10), we found that simultaneous injection of both types of NPs ameliorates the course of EAE in both prophylactic and therapeutic vaccination. By contrast, immunization with only one type of these NPs (either PLGA-MOG or PLGA-IL10) did not have any effect. The positive effect on the clinical features of the disease was paralleled by decreased inflammation and T-cell infiltration in the CNS and decreased production of the proinflammatory cytokines IL-17 and IFN-γ induced by stimulating T cells in vitro with MOG_35–55_ [58]. In another study, Maldonaldo and co-workers developed PLGA-NPs loaded with the immunodominant 139–151 epitope of PLP (PLP_139–151_) in SJL mice together with rapamycin [90], used as inverse adjuvant, and administrated them i.v. into an RR model of EAE. Prophylactic treatment using these NPs inhibited the onset of EAE, whereas the therapeutic treatment inhibited relapse. Intriguingly, PLGA-NPs containing only PLP_139–151_ had a partial tolerogenic effect, which might be ascribed to the i.v. administration of this vaccine, which probably triggered higher levels of deletional tolerance than those seen after s.c. injection [90]. In support of this hypothesis, Getts et al. showed that i.v. but not s.c. injections of PLGA-NPs or polystyrene beads covalently linked to MOG_35–55_ on their surface display a protective effect in RR-EAE using both prophylactic and therapeutic treatments in the absence of inverse adjuvants, an effect mainly due to deletional tolerance [91]. 

The different requirements of tolerogenic vaccines delivered through the i.v. or s.c. routes have been nicely addressed by Casey et al [92], who studied the tolerogenic properties of PLGA-NPs loaded with PLP_139–151_ and chemically coupled to TGF-β1 on their surface (PLGA_PLP139–151_-TGF-β-NPs). In preliminary experiments, these NPs were found to reduce the expression of costimulatory molecules (i.e., CD80 and CD86) in immature and mature bone marrow DCs in vitro. Working on an RR model of EAE induced with PLP_139–151_, these authors compared the tolerogenic activity of PLGA_PLP139–151_-TGF-β NPs with that of PLGA-NPs loaded with PLP_139–151_ in the absence of TGF-β-(PLGA_PLP139–151_-NPs) following either i.v. or s.c. administration. Results showed that both types of NPs ameliorated EAE symptoms when administrated by i.v. injections, whereas only PLGA_PLP139–151_-TGF-β NPs were effective upon s.c. administration. Working on the anti-OVA response in OT-II TCR transgenic mice (which are transgenic for an anti-OVA TCR), the same lab also showed that surface binding of TGF-β to PLGA-NPs loaded with OVA is required to efficiently induce tolerance to OVA in an antigen-specific manner [92]. These findings indicate that s.c. inverse vaccination needs inverse adjuvants in order to be effective.

The amount of antigen conjugated to NPs and the NP dose are additional key features for induction of immune tolerance, as shown by Kuo et al [93]. In this study, a single i.v. injection of high-dose PLGA-NPs carrying high amounts of PLP_139–151_ significantly decreased the severity of RR-EAE, and no relapses were observed. By contrast, i.v. injection of either low-dose PLGA-NPs carrying high amounts of PLP_139–151_ or high-dose PLGA-NPs carrying low amounts of PLP_139–151_ did not lead to durable tolerance, and the treated mice experienced relapses. Interestingly, high-dose PLGA-NPs loaded with high amounts of PLP_139–151_ substantially decreased DC expression of co-stimulatory molecules involved in activating effector T cell function [93]. 

PLGA-NPs can also be loaded with metabolic modulators that alter DC functions and promote shifting of T cells from the Th17 to the Treg phenotype [94]. Another approach is represented by the development of dual-sized NP/MPs platform comprising small phagocytosable particles delivering the antigen and other intracellularly active agents and large unphagocytosable particles releasing factors capable of modulating DC functions. A dual-sized PLGA platform encapsulating MOG_35–55_ and vitamin D in the phagocytosable small particles, and TGF-β and GM-CSF in the large non-phagocytosable particles was effective in treating EAE using a semi-therapeutic regimen, with an antigen-specific effect [95]. 

Tolerogenic PLGA-NPs can also function as direct modulators of autoreactive T cells without eliciting the intervention of tolerogenic DCs. A recent study described PLGA-NPs encapsulating TGF-β and covered on the surface with three following types of molecules: (i) multimers of MHC class I and II molecules loaded with myelin peptides to target autoreactive T cells, (ii) anti-Fas mAb alongside a recombinant PD-L1-Fc construct, capable of inducing apoptosis or dysfunction of the autoreactive T cells bound to the MHC multimers, and (iii) CD47-Fc able to inhibit NP phagocytosis, thereby prolonging their activity. These NPs directly inhibited the myelin-autoreactive T cells without requiring uptake, processing, and presentation by APCs. Intravenous infusion of these multivalent NPs durably ameliorated EAE with a marked reduction in clinical, neuroinflammation, and demyelination scores [96].

Finally, a novel optical imaging technique was used to quantify the brain and spinal cord alterations in mice treated intravenously with PLP_139–151_ chemically coupled to PLGA-NPs in a RR-EAE model. This study used imaging agents able to cross the blood brain barrier and showed that PLGA-NPs effectively inhibited both the onset of the disease and the accumulation of imaging agents into the brain and spinal cord [105]. 

#### 3.2.2. Rheumatoid Arthritis

Rheumatoid arthritis (RA) is an autoimmune disease characterized by progressive destruction of the articular cartilage [106]. Although the etiology of RA remains unclear, type II collagen (CII) is generally considered a potential RA-associated autoantigen due to its relative abundance in the cartilage, which is an immune privileged tissue [107]. In support of this hypothesis, immunization with CII has been shown to induce an RA-like disease, named collagen-induced-arthritis (CIA), in susceptible strains of mice and rats [108]. Furthermore, Kim et al. showed that a single oral feeding with PLGA-NPs encapsulating CII in DBA/1 mice protects these mice from CIA [97]. Indeed, CII was retained up to 14 days in the dome area of Peyer’s patches, where oral tolerance usually takes place. Similarly, nasal delivery of the antigen could induce antigen-specific tolerance not only locally but also in distant peripheral mucosal tissues [109]. Furthermore, Keijzer et al. showed that nasal vaccination with PLGA-NPs encapsulating the protein HSP70, which has suppressive properties, is able to reduce arthritis severity in the proteoglycan-induced arthritis (PGIA) mouse model. Finally, nasal vaccination with PLGA-NPs entrapping OVA resulted in suppression of a Th1-mediated hypersensitivity reaction against OVA in mice [98].

#### 3.2.3. Type 1 Diabetes

T1D is an autoimmune disease targeting insulin-producing β cells of the pancreatic islets and is mainly caused by autoreactive T cells recognizing several β cell autoantigens [110]. 

Yoon et al., incorporated PLGA microparticles loaded with insulin in PuraMatrix^TM^ peptide hydrogel containing GM-CSF and CpG sequences (CpG ODN1826), used to recruit and activate immune cells [99]. In this formulation, PLGA was used as a scaffold to allow infiltration of the target immune cells into the material [111]. Subcutaneous administration of this hydrogel prevented development of T1D in NOD mice, a mouse model of spontaneous T1D. Splenocytes from hydrogel-treated mice led to production of high level of IL-10 supporting tolerogenic DCs and Tregs. Moreover, multiple subcutaneous injections of this hydrogel caused formation of granulomas consisting of infiltrated immune cells, indicating the creation of a microenvironment responsible for the recruitment of immune cells and the regulation of their functions [99].

Hydrogels consist of 3-D macromolecular polymeric chains that can be easily molded in any form, shape, and size and be engineered to mimic the extracellular environment of the body’s tissue. They do not dissolve and can absorb high amounts of water, which can amount to thousand folds of their own dry weight, allowing controlled release of the drugs [112]. The effectiveness of this approach was confirmed by another study using a scaffold hydrogel consisting of PLGA-NPs loaded with the BDC peptide [100]. This peptide acts as an antigen mimotope recognized by autoreactive BDC2.5 T cells in NOD mice—incorporated in alginate hydrogel loaded with GM-CSF. Upon s.c. administration to NOD mice, the majority of cells accumulating in the gel were Tregs, and these hydrogels tended to delay progression of T1D, but the effect was not significant [100]. 

Finally, s.c. injection of a dual-sized PLGA platform consisting of phagocytosable particles (encapsulating vitamin D3 or insulin B_(9–23)_ peptide) and unphagocytosable particles, encapsulating TGF-β1 or GM-CSF, significantly protected mice from T1D development [101].

## 4. Conclusions

Current treatments for autoimmune diseases are not antigen-specific and mostly rely on the use of immunosuppressive agents, which can lead to unspecific immunosuppression and increased risk of infections and cancer. In contrast, tolerogenic vaccines are designed to dampen aberrant immune responses against self-antigens while preserving the immune response against foreign antigens and pathogens. 

In this scenario, PLGA-NP-based tolerogenic vaccines have emerged as powerful tools to re-establish immunological tolerance, thereby preventing autoimmune disease. Given that PLGA is already approved for human use by the FDA, the possibility that the encouraging results obtained on animals using PLGA-NPs can be quickly translated to humans seems quite real. The effectiveness of PLGA-based inverse vaccination in several autoimmune disease mouse models represents a proof-of-concept that this approach is feasible, which could be extended to other experimental diseases and other NP platforms. In light of the reported literature, it is likely that the choice of different NP materials will be crucial to modulate the vaccine capacity toward different types of immunological response. 

A key problem in translating reverse vaccination to humans is that in human autoimmune diseases the autoimmune response is generally heterogeneous. Furthermore, each disease can involve multiple autoantigens that can be different in different patients depending on the genetic background, age, environmental and triggering factors, and disease duration. Moreover, in chronic autoimmune disease the pattern of autoantigens recognized gradually increases during the disease course, a phenomenon known as epitope spreading. Therefore, identifying which autoantigens should be included in the tolerogenic vaccine could be quite challenging. This problem may be partly overcome by tolerance spreading, that is the gradual spread of the tolerance to the autoantigens included in the vaccine to other autoantigens involved in autoimmunity.

An alternative approach could consist in using NPs to deliver immunosuppressive substances (e.g., methotrexate and lovastatin) into the tissue targeted by the autoimmune disease, without including autoantigens in the NPs. This approach may be envisaged as a potential tolerogenic vaccination where the autoantigens are already present in the tissue, and the immunosuppressive agent functions locally as inverse adjuvant breaking the autoimmune circuits within the damaged tissues. For example, i.v. injection of multifunctional PEG-PLGA NPs (composed of lipids, folic acid and poly (cyclohexane-1,4-diylacetone dimethylene ketal)) entrapping methotrexate allowed the direct release of methotrexate into inflamed tissue and suppressed adjuvant-induced arthritis [113]. In another study, methotrexate was encapsulated in PLGA (Au)/iron (Fe)/gold (Au) half shell NPs conjugated with αvβ3 integrin, able to bind RGD amino acid sequences, in order to increase tissue uptake. Upon near-infrared (NIR) irradiation, the local heat generated by the NIR resonance of the Au half-shells leads to release of methotrexate from the PLGA-NPs into the inflamed tissue [114]. Scheinman et al. developed PLGA-NPs entrapping STAT1 siRNA and functionalized with tripeptide Arg-Gly-Asp (RGD) motifs binding to integrins. Local delivery of these NPs into the joints decreased expression of STAT1, induced regression of established arthritis in the CIA model, and increased production of IL-10 [115]. Finally, bilateral perineural administration of PLGA NPs loaded with lovastatin significantly attenuated clinical severity of experimental autoimmune neuritis (EAN), a model of Guillain-Barré syndrome and chronic inflammatory demyelinating polyradiculoneuropathy, and protected animals from peripheral nerve morphological and functional deficits [116]. 

In conclusion, nanotechnology is an evolving and rapidly growing technology worldwide. The relatively easy manufacturing process for PLGA-NPs and their low cost make them rather attractive for the vaccine market. Up to date, there are 15 FDA-approved PLA/PLGA-based drug products available on the US market, and they are mainly composed of anti-cancer-agents [117]. 

## Abbreviations

AFM	Atomic force microscope
APC	Antigen-presenting cell
BSA	Bovine serum albumin
CFA	Complete Freund’s adjuvant
CIA	Collagen-induced arthritis
CII	Collagen type II
CNS	Central nervous system
DC	Dendritic cells
EAE	Experimental autoimmune encephalomyelitis
EAN	Experimental autoimmune neuritis
EPR	Enhanced permeability and retention
FDA	Food and drug Administration
GM-CSF	Granulocyte-macrophage colony-stimulating factor
HSP	Heat shock protein
i.v.	intravenous
IFN	interferon
Ig	immunoglobulin
IL	interleukin
iTreg	Induced Treg
MBP	Myelin basic protein
MHC	Major histocompatibility complex
MOG	Myelin-oligodendrocyte glycoprotein
MS	Multiple sclerosis
NIR	Near-infrared
NK	Natural killer
NOD	Non Obese Diabetic
NPs	nanoparticles
nTreg	Natural Treg
OVA	Ovalbumin
PEG	Polyethylene glycol
PGIA	Proteoglycan-induced arthritis
PLGA	Poly(lactic-co-glycolic acid)
PLP	Proteolipid protein
RA	rheumatoid arthritis
RR	Relapse remitting
s.c.	subcutaneous
SEM	Scanning electron microscopy
T1D	Type 1 diabetes
TCR	T-cell receptor
TEM	transmission electron microscope
TFH	T follicular helper
TGF	Transforming growth factor
Th	T helper
TLR	Toll like receptor
Treg	T regulatory
